# Sustainable β-carotene production from sorghum syrup using *Rhodotorula glutinis*: bioprocess optimization and scale-up

**DOI:** 10.1093/jimb/kuaf032

**Published:** 2025-10-28

**Authors:** Kevaughn Prout, Keerthi Mandyam, Ananda Nanjundaswamy

**Affiliations:** Department of Biology and Microbiology, South Dakota State University, Brookings, SD, USA; Department of Agronomy, Horticulture and Plant Science, South Dakota State University, Brookings, SD, USA; Department of Biology and Microbiology, South Dakota State University, Brookings, SD, USA

**Keywords:** RSM, bioprocessing, carotenoids, red-yeasts, valorization

## Abstract

β-Carotene, a key provitamin A carotenoid, is widely used as an antioxidant and natural pigment. Due to animals' inability to synthesize carotenoids, dietary sources are essential. This study utilized low-cost sorghum syrup for β-carotene production via *Rhodotorula glutinis* fermentation. Bioprocess optimization using response surface methodology was conducted in shake flasks, then scaled to 300 mL and 7 L fermentations. The optimized medium (9.18% sorghum syrup, 0.96% yeast extract, 0.07% KH₂PO_4_, 0.13% (NH_4_)₂SO_4_, 0.42% MgSO_4_) yielded a predicted 1 003 µg/g β-carotene after 10 days. Scale-up achieved 1 153 µg/g (300 mL) and 1 753.33 µg/g (7 L). Nutritional analysis showed the presence of chelated minerals, vitamins, proteins, and glucosamine, enhancing biomass value. These results highlight sorghum syrup as an effective, sustainable substrate for β-carotene production with applications in food, feed, and nutraceutical sectors.

**One Sentence Summary**: Using sorghum syrup as a low-cost substrate, we optimized β-carotene production *by Rhodotorula glutinis* via response surface methodology and validated at 7 L scale (up to 1,753 μg/g), while profiling the nutrient-dense biomass (protein, minerals, glucosamine) for food/feed applications.

## Graphical Abstract

**Graphical Abstract:**
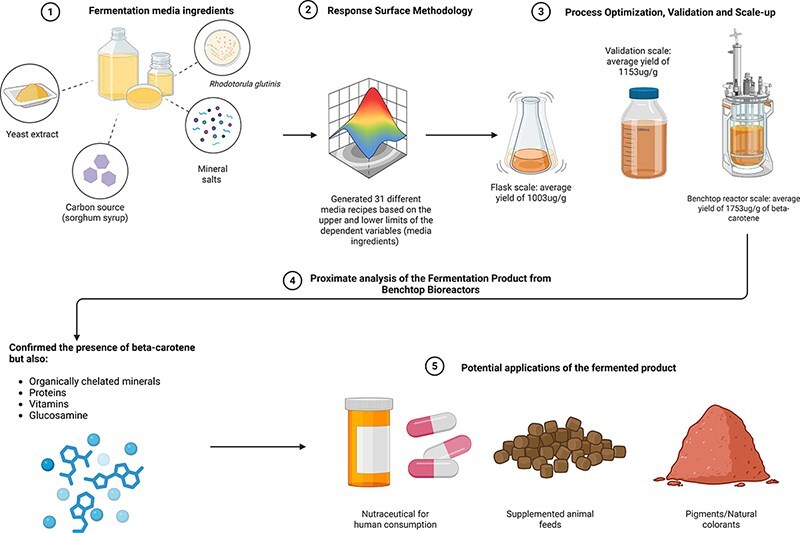

## Introduction

Carotenoids are a diverse group of lipophilic pigments responsible for the red, orange, and yellow hues observed across plants, algae, fungi, and bacteria (Ashokkumar et al., 2023; Rodriguez-Amaya, 2019; Sandmann, 2022). To date, more than 1 200 naturally occurring carotenoids have been identified and characterized (Yabuzaki, 2017), including β-carotene, torulene, astaxanthin, and torularhodin (Rapoport et al., 2021). Beyond their role as pigments, carotenoids possess strong antioxidant properties, functioning by quenching reactive oxygen species, thereby providing protective health benefits (Di Mascio et al., 1991; Fiedor & Burda, 2014).

Although synthetic carotenoids are widely available, their production relies on petrochemical processes that raise environmental and health concerns, including potential carcinogenicity (Lourenço-Lopes et al., 2021). Growing consumer demand for natural ingredients, driven by chemophobia and health-conscious practices, has increased interest in sustainably produced natural carotenoids (Dufossé et al., 2005).

The global carotenoids market was valued at approximately USD 2 billion in 2022, with an expected compound annual growth rate of 5.7% from 2023 to 2028, projected to reach USD 2.7 billion by 2027 (Global Carotenoids Market Report and Forecast 2023–2028, n.d.). β-Carotene, the most studied and commercially important carotenoid, alone accounted for USD 261 million in 2010 and USD 334 million in 2018, highlighting its wide applicability and growing demand.

To reduce production costs and improve sustainability, recent efforts have focused on utilizing agro-industrial byproducts as substrates for microbial carotenoid production (Aksu & Eren, 2005; Sharma & Ghoshal, 2020; Thumkasem et al., 2024). Sweet sorghum (*Sorghum bicolor* (L.) Moench) is an attractive feedstock option due to its wide geographic adaptability, short growing cycle (3–5 months), low water and fertilizer requirements, and resilience to drought and temperature fluctuations (Teetor et al., 2011). Sorghum syrup, derived from stalk juice, is rich in sugars, vitamins, iron, potassium, and magnesium, making it a cost-effective, nutrient-rich substrate for microbial cultivation (Eggleston et al., 2022).

Among microbial carotenoid producers, *Rhodotorula* species, particularly *R. glutinis*, have gained attention for their ability to synthesize carotenoids, enzymes, and microbial oils such as oleic, linoleic, palmitic, and stearic acids (Grigore et al., 2023; Kot et al., 2016). These yeasts exhibit rapid growth, utilize diverse carbon sources, and require minimal cultivation inputs compared to plant- or algae-based systems (Kim et al., 1999; Venil et al., 2013).

Process optimization, including response surface methodology, has been successfully employed to enhance microbial carotenoid production (Ananda & Vadlani, 2011; Bhosale, 2004). Coupling such approaches with low-cost, agricultural substrates like sorghum syrup offers an economically viable strategy for natural β-carotene production.

The present study investigated the feasibility of using sorghum syrup as the primary carbon source for β-carotene production by *R. glutinis*. We hypothesized that sorghum syrup could support both high carotenoid yields and provide additional nutritional value in the final product. The objectives were to (i) optimize β-carotene production in *R. glutinis* using sorghum syrup-based media (ii) Scale-up the bioprocessing to benchtop bioreactors and (iii) evaluate the nutritional/biochemical composition of the fermented product.

## Materials and Methods

### Chemicals and Reagents

All chemicals and reagents used in this study were of analytical grade and purchased from Fisher Scientific. HPLC-grade acetonitrile and methanol were used for chromatographic analysis. The β-carotene standard was obtained from Millipore-Sigma.

### Microorganism and Growth Conditions

*R. glutinis* (ATCC 32766) used in this study was obtained from the American Type Culture Collection (ATCC, Manassas, VA, USA). The strain was cultivated in 250 mL Erlenmeyer flasks containing 100 mL of sterile potato dextrose broth (PDB) and incubated at 25 °C with agitation at 200 rpm for 7 days. The culture was also maintained on potato dextrose agar (PDA) plates at room temperature for routine use. The broth culture was subsequently used as the inoculum for all experimental evaluations.

### Process Optimization

A central composite design (CCD) was employed to optimize the key medium components for β-carotene production. Five independent variables were evaluated: sorghum syrup (1–10%, 100% pure, Muddy Pond Sorghum Mill, Monterey, TN, USA), ammonium sulfate [(NH_4_)₂SO_4_, 0.01–0.1%], potassium phosphate (KH₂PO_4_, 0.01–0.1%), magnesium sulfate (MgSO_4_, 0.01–0.1%), and yeast extract (0.1–1%, Research Products International, Mt. Prospect, IL, USA).

Design-Expert® software version 13 (Stat-Ease Inc., Minneapolis, MN, USA) was used to generate the experimental matrix, analyze the influence of the independent variables on β-carotene production and biomass yield, and develop response surface and contour plots. A design matrix with minimum (-1) and maximum (+1) is provided in Table 1. A detailed 31 experimental matrix is given in Table S1.

**Table 1.** Media composition for RSM with minimum (-1) and maximum (+1) for all selected ingredients

Factor	A	B	C	D	E
Name	Sorghum syrup	(NH_4_)_2_SO_4_	KH_2_PO_4_	MgSO_4_	Yeast extract
Units	%	%	%	%	%
Type	Numeric	Numeric	Numeric	Numeric	Numeric
Subtype	Continuous	Continuous	Continuous	Continuous	Continuous
Minimum	1.0000	0.0500	0.0100	0.1000	0.1000
Maximum	10.00	0.5000	0.1000	0.5000	1.0000
Coded low	-1 ↔ 1.00	-1 ↔ 0.05	-1 ↔ 0.01	-1 ↔ 0.10	-1 ↔ 0.10
Coded high	+1 ↔ 10.00	+1 ↔ 0.50	+1 ↔ 0.10	+1 ↔ 0.50	+1 ↔ 1.00
Mean	5.37	0.2900	0.0569	0.2904	0.5411
Std. Dev.	3.64	0.1783	0.0356	0.1606	0.3739

The CCD was conducted to determine the optimal conditions for β-carotene production at the shake flask level, assessing both the individual and interactive effects of the media components on yeast growth and pigment synthesis. Experiments were performed in 250 mL Erlenmeyer flasks containing 100 mL of working volume. The flasks were sterilized at 120 °C for 30 minutes and cooled to room temperature before inoculation with 1 mL of an actively growing *R. glutinis* culture. Flasks were incubated at 25 °C with shaking at 200 rpm for 10 days.

At the end of fermentation cultures were centrifuged, the supernatant discarded, and the cell pellets freeze-dried for β-carotene quantification. Freeze drying was carried out at -80°C at pressure 2.6 × 10^–4^ psi using Labconco benchtop freeze-dryer.

The relationship between independent variables and responses was modeled using a second-order (quadratic) polynomial equation (Equation 1).


(1) \begin{eqnarray*}
y &=& 2{{x}_1}^2 - 3{{x}_2}^2 + 4{{x}_3}^2 + {{x}_4}^2 - 5{{x}_5}^2 + 3{{x}_1}{{x}_2} - 2{{x}_2}{{x}_3} + {{x}_3}{{x}_4} \\
&& + 2{{x}_4}{{x}_5} + 5{{x}_1} - {{x}_2} + 3{{x}_3} + 4{{x}_4} - 2{{x}_5} + 7
\end{eqnarray*}

Where ***y*** is the dependent variable and x_1_ = sorghum syrup, x_2_= (NH_4_)_2_SO_4_, x_3_ = KH_2_PO_4_ x_4_ = MgSO_4_ and x_5_ = Yeast extract, x^2^_i_ represent the squared influence of each variable, *x_i_x_j_* capture how pairs of variables interact, x_i_ represent direct, proportional effects, 7 represents the constant term and is the baseline value of y when all x*_i_* = 0

### Carotenoid Extraction and Quantification

Carotenoids were extracted from approximately 0.2 g of freeze-dried *R. glutinis* cells using 10 mL of tetrahydrofuran. The extraction was performed by grinding the cells with acid-washed sand in a mortar and pestle to facilitate cell disruption and pigment release. The extracts were filtered through 0.2 µm syringe filters into HPLC vials for analysis (Ananda and Vadlani, 2011).

Shimadzu High-performance liquid chromatography (HPLC) equipped with LC-40D HPLC Pump, DGU-405 5-channel Degasser, SPD-M40 Photodiode Array Detector, SIL-40C auto-sampler and CTO-40C Column Oven, CBM-40 System Controller and LabSolutions software was used for analysis. A method described in Ananda and Vadlani (2011) was used with slight modification. Carotenoid separation was achieved using a Phenomenex Luna C8 column (150 mm × 4.6 mm), with a mobile phase consisting of acetonitrile and methanol (90:10, v/v) at a flow rate of 1.5 mL/min. Detection was carried out at 450 nm, and β-carotene concentrations were quantified using a calibration curve prepared with a pure β-carotene standard (Millipore-Sigma, St. Louis, MO, USA). A sample chromatogram extracted at 455 nm is given in Figure S1.

### Validation of Optimized Medium in Media Bottles

The optimal medium composition determined through the central composite design (CCD) was used to prepare 2 liters of fermentation medium. Aliquots of 300 mL were dispensed into sterile one liter glass media bottles in duplicates and inoculated with 1% (v/v) of actively growing *R. glutinis* culture. All bottles were incubated on an orbital shaker at 25 °C and 200 rpm for 10 days. Samples were collected from each bottle on days 5, 7, and 10, and β-carotene content was quantified using HPLC as described previously.

### Validation in Benchtop Bioreactors

A batch-mode fermentation was conducted using three 7 L benchtop bioreactors (BioFlo® 115, Eppendorf AG, Germany), each operated in triplicate. The working volume for each bioreactor was 5 L, prepared with the optimized medium composition consisting of 459 g sorghum syrup, 6.5 g (NH_4_)₂SO_4_, 3.5 g KH₂PO_4_, 21 g MgSO_4_, and 48 g yeast extract. Following sterilization, the bioreactors were inoculated with 1% (v/v) of a 48-hour-old *R. glutinis* culture (Figure S2).

Fermentation was carried out under controlled batch-mode conditions: temperature at 25 °C, agitation at 200 rpm, and aeration maintained at 1 vvm. All bioreactors were integrated with the BioCommand® supervisory control and data acquisition (SCADA) system, which collected real-time data from each unit at 10-minute intervals over the 10-day fermentation period. Samples were drawn on day 5, day 7 and harvest day of 10. Samples were centrifuged and yeast cell pellets were freeze dried. Freeze dried samples were used for β-carotene estimation. The day 10 freeze dried samples were also used for nutritional characterization. The fermentation profiles, including pH, dissolved oxygen (DO), temperature, and agitation speed, were continuously monitored and recorded.

### Nutritional Characterization of the Fermented Product

At the end of the 10-day fermentation, the culture broth was harvested by centrifugation, and the resulting biomass was stored at –80 °C. All biomass samples from the triplicate bioreactors were freeze-dried and analyzed for nutritional composition, including vitamins (riboflavin, niacin, pantothenic acid, and pyridoxine), total amino acids, total fatty acids, crude fat, crude protein, crude fiber, glucosamine, and minerals. B vitamins were quantified using the LC-MS/MS method described by the (Viñas et al 2003), while vitamin D and vitamin E were analyzed using AOAC Methods 2016.05 and the *CRNFS* (2016, Vol. 4, p. 92) method, respectively. Crude protein and crude fat were determined by AOAC Methods 990.03 and 2003.05, and total amino acids were measured using AOAC Methods 994.12 and 988.15. Total lipids and fatty acids were analyzed following AOAC Method 996.06, glucosamine was assessed using the (Pastorini et al. 2011), and mineral content was determined using AOAC Method 985.01.

### Statistical Analysis

The statistical analysis was conducted using Statistical Analysis Software (SAS, version 9.4, Cary, NC). PROC ANOVA was used to compare the treatments and Tukey pairwise differences was used. For correlation, PROC CORR was used. Significance was set at P = 0.05.

## Results and Discussion

### Optimizing Culture Medium for β-carotene Production

**Fig. 1.**
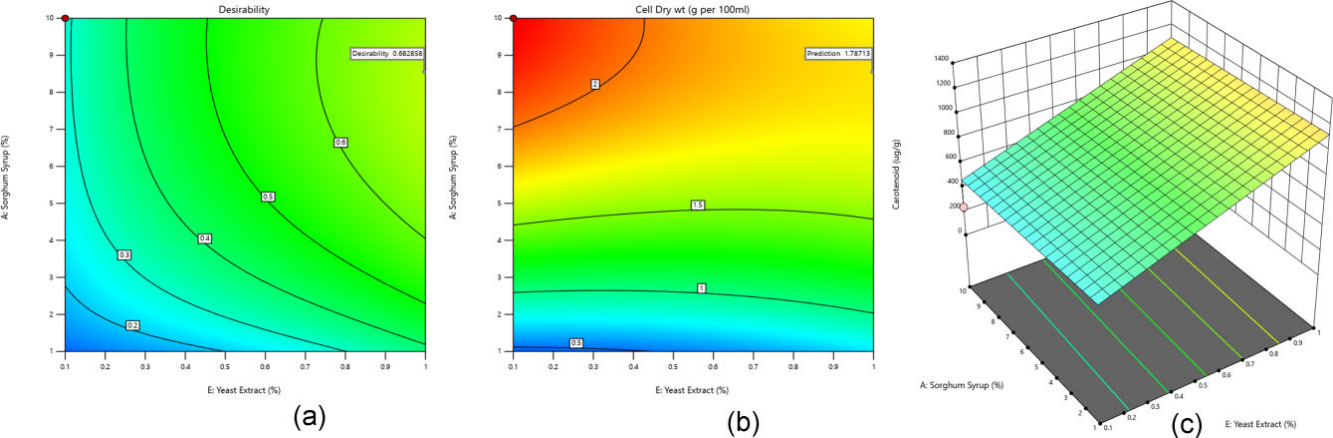
Contour plots illustrating the effects of media components sorghum syrup and yeast extract on: (a) desirability of the interaction b. Cell dry wt. of R. glutinis and c. β-carotene concentration.

**Fig. 2.**
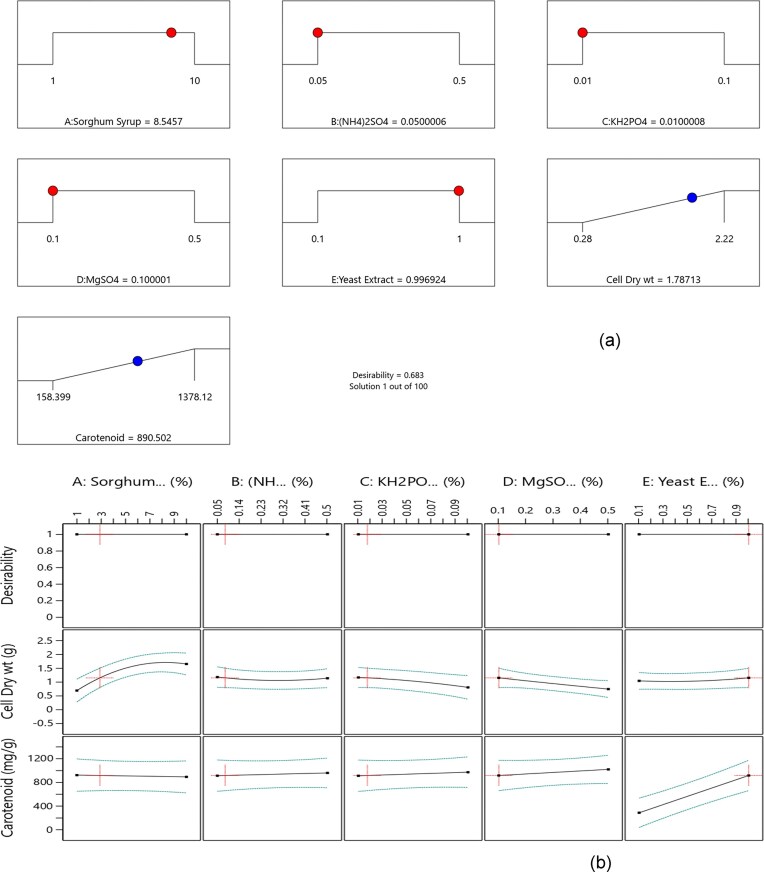
(a) RSM plot outlining the optimal points for the media components for β-carotene production in R. glutinis (9.18% sorghum syrup, 0.13% (NH_4_)_2_SO_4_, 0.07% KH_2_PO_4_, 0.42% MgSO_4_ and 0.96% yeast extract). (b) StatEase-generated RSM interaction plot depicting

Bioprocess optimization using a central composite design (CCD) with 31 experimental combinations of sorghum syrup-based culture media revealed the optimal conditions for β-carotene production by *R. glutinis*. The β-carotene yields and percent dry cell weight for all experimental runs were summarized in Table S2. β-Carotene production ranged from 158 µg/g to 1 378 µg/g of dry cell weight, while dry cell weight ranged from 0.3% to 2.22%. The contour plots in Fig. 1 illustrated the interaction between sorghum syrup and yeast extract concentrations and their influence on overall desirability keeping mineral concentrations at 0.07% KH₂PO_4_, 0.13% (NH_4_)₂SO_4_, and 0.42% MgSO_4_, (Fig. 1a), dry cell weight (Fig. 1b), and β-carotene production (Fig. 1c). The results of the Analysis of Variance (ANOVA) for the response surface model were presented in Table 2. The model was statistically significant (p < 0.0002), and the lack-of-fit was non-significant (F = 1.44, p = 0.366), indicating that the model adequately described the experimental data. The optimization results showed that achieving both high cell biomass and β-carotene production required a balance between carbon and nitrogen sources. Although a desirability of 0.68 was achieved for dry cell weight (1.78% dry weight), the maximum predicted β-carotene yield of 1 003.38 µg/g of dried yeast cells was not fully attained under those conditions, indicating a trade-off between biomass accumulation and pigment production (Fig. 2a).

**Table 2.** ANOVA for response surface method (RSM) model for variables in the study

Source	Sum of squares	df	Mean square	F-value	*P*-value	
**Model**	2.26E + 06	5	4.53E + 05	7.54	0.0002	significant
A-sorghum syrup	4357.14	1	4357.14	0.0726	0.7898	
B-(NH_4_)_2_SO_4_	9816.29	1	9816.29	0.1635	0.6894	
C-KH_2_PO_4_	17 050.31	1	17 050.31	0.284	0.5988	
D-MgSO_4_	48 711.35	1	48 711.35	0.8115	0.3763	
E-yeast extract	1.96E + 06	1	1.96E + 06	32.64	< 0.0001	
**Residual**	1.50E + 06	25	60 029.94			
Lack of fit	1.28E + 06	20	63 927.48	1.44	0.3668	not significant
Pure error	2.22E + 05	5	44 439.78			
**Cor total**	3.77E + 06	30				

The data further demonstrated that an increase in sorghum syrup concentration positively influenced cell biomass production, while an increase in yeast extract concentration enhanced β-carotene yields (Fig. 2b). These trends were in contrast with earlier findings, where nitrogen limitation stimulated carotenoid biosynthesis, while adequate nitrogen levels promoted cell growth (Anzola-Rojas et al., 2015; Mata-Gómez et al., 2014; Singh et al., 2020). Sharma and Ghoshal (2020) also observed improved biomass and carotenoid production in *Rhodotorula mucilaginosa* when agricultural waste hydrolysates with higher sugar concentrations were utilized. It was well established that the carbon-to-nitrogen (C/N) ratio regulated carotenoid biosynthesis in oleaginous yeasts such as *Rhodotorula* spp. Ratios near 20:1 favored carotenoid accumulation by channeling acetyl-CoA toward the mevalonate pathway, while higher C/N ratios (>50:1) promoted fatty acid synthesis as a cellular response to excess carbon availability (Paul, Bohacz, et al., 2023, 2023; Tkáčová et al., 2022). In this study, the optimized medium contained approximately 9.18% sorghum syrup (carbon source) and 0.96% yeast extract (nitrogen source), corresponding to an estimated C/N ratio of ∼20:1 to 25:1, which supported carotenoid production. Detailed concentrations of the optimized media components and their corresponding desirability values were provided in Table S3 (Supplementary Data). The correlation between the predicted and experimental values for β-carotene yield and dry cell weight was illustrated in Figure S3. The regression model demonstrated a strong agreement between predicted and experimental dry cell weight values, while β-carotene yields exhibited higher variability, likely due to the sensitivity of carotenoid biosynthesis to subtle changes in media composition. The final regression models representing the combined effects of the independent variables on β-carotene yield and dry cell weight were provided in Equations 2 and 3, respectively.


(2) \begin{eqnarray*}
&&{\bf \textit{Carotenoid}} = + 182.25203 - 3.32468\, \textit{Sorghum}\textit{Syrup} \\
&& \quad + 102.71735{{\left( {N{{H}_4}} \right)}_2}S{{O}_4} + 674.83896K{{H}_2}PO4 \\
&& \quad + 257.89752MgS{{O}_4} + 701.14336\textit{Yeast}
\end{eqnarray*}

(3) \begin{eqnarray*}
&&{\mathrm{Cell\ Dry\ wt}}{\mathrm{.}} = + 0.201416 + 0.435647{\bf }\textit{Sorghum} \textit{Syrup} \\
&& \,\,\,\,- 1.32862\left( {N{{H}_4}} \right)2S{{O}_4} - 0.575879K{{H}_2}P{{O}_4} \\
&& \,\,\,\,- 0.934599MgS{{O}_4} + 0.068443\textit{Yeast}{\bf }\textit{Extract} \\
&& \,\,\,\,- 0.136133\textit{Sorghum}{\bf }\textit{Syrup}*{{\left( {NH4} \right)}_2}SO4 \\
&& \,\,\,\,- 0.269135\textit{Sorghum}{\bf }\textit{Syrup}*K{{H}_2}P{{O}_4} \\
&& \,\,\,\,- 0.077574\textit{Sorghum}{\bf }\textit{Syrup}*MgS{{O}_4} - 0.094919\textit{Sorghum}{\bf }\textit{Syrup} \\
&& \,\,\,\, * \textit{Yeast}{\bf }\textit{Extract} - 1.42213\left( {N{{H}_4}} \right)2S{{O}_4}\\
&& \,\,\,\, *K{{H}_2}P{{O}_4} + 0.464502\left( {N{{H}_4}} \right)2S{{O}_4}\\
&& \,\,\,\, * MgS{{O}_4} + 0.506129\left( {N{{H}_4}} \right)2S{{O}_4}*\textit{Yeast}\ \textit{Extract} \\
&& \,\,\,\, + 19.22349K{{H}_2}P{{O}_4}*MgS{{O}_4} - 1.83830K{{H}_2}P{{O}_4}\\
&& \,\,\,\, * \textit{Yeast}{\bf }\textit{Extract} - 0.308211MgS{{O}_4}*\textit{Yeast}\ \textit{Extract} \\
&& \,\,\,\, - 0.019160\textit{Sorghum}\ \textit{Syru}{{p}^2} + 2.00214{{\left( {{{{\left( {N{{H}_4}} \right)}}_2}S{{O}_4}} \right)}^2} \\
&& \,\,\,\, - 23.58884{{\left( {K{{H}_2}P{{O}_4}} \right)}^2} + 0.142976{{\left( {MgS{{O}_4}} \right)}^2}\\
&& \,\,\,\, + 0.316454\textit{Yeast}{\bf }\textit{Extrac}{{t}^2}
\end{eqnarray*}

Despite the low concentrations of minerals present in the fermentation medium, their specific levels appeared to influence β-carotene production. While the precise role of inorganic salts in carotenogenesis remains not fully understood, previous studies have demonstrated that the addition of minerals to the culture medium can enhance carotenoid yields (Han et al., 2002). It is plausible that minerals play a critical role in regulating enzyme activities involved in carotenogenic pathways.

In this study, mineral salts were observed to have a positive influence on β-carotene production but a negative effect on cell biomass (Fig. 2b). Similar trends have been reported in the literature. For example, Chen et al. (2006) demonstrated enhanced carotenoid production in *Rhodotorula sphaeroides* cultured in media supplemented with MgSO_4_, Na₂HPO_4_, FeSO_4_, and Na₂CO₃. reported that KH₂PO_4_ and MgSO_4_ were essential for β-carotene synthesis in *Blakeslea trispora*. Likewise and Parajó et al. (1998) showed that *Phaffia rhodozyma* produced astaxanthin under nitrogen-limited conditions when KNO₃ and (NH_4_)₂SO_4_ were used as nitrogen sources which stimulated β-carotene production.

In alignment with these findings, the present study incorporated (NH_4_)₂SO_4_ as the primary inorganic nitrogen source, supplemented by organic nitrogen sources from yeast extract and sorghum syrup. The optimization experiments revealed that higher yeast extract concentrations significantly enhanced β-carotene production, while higher concentrations of sorghum syrup primarily increased cell biomass (dry cell weight) of *R. glutinis*. It is possible that sorghum syrup, being rich in naturally occurring minerals, reduced the requirement for additional inorganic mineral supplementation during fermentation, particularly for carotenoid biosynthesis.

### Batch-mode Validation in Media Bottles and Benchtop Bioreactors

**Fig. 3.**
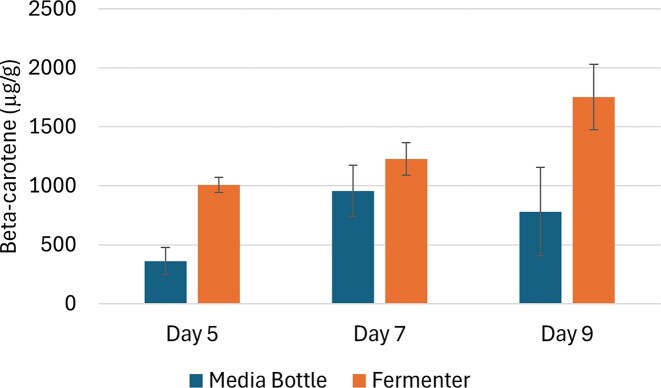
Validation experiment in media bottles and benchtop bioreactors using the optimal points obtained from RSM. Samples from two replicates per time point were pooled before analysis (x axis-days, y axis-yield µg/g).

Validation experiments using media bottles further confirmed the influence of fermentation duration on β-carotene yields (Fig. 3). β-Carotene concentrations on days 5, 7, and 10 were 476, 1 173, and 1 153 µg/g of dry cell weight, respectively. Interestingly, the day 10 yield of 1 153 µg/g slightly exceeded the predicted yield of 1 003.38 µg/g, confirming the accuracy of the response surface model. However, the highest yield was recorded on day 7, surpassing both the predicted and final day 10 yields. There was no statistically significant difference among days with respect to β-carotene production is concern, but highest was noticed on day 10.

These media bottle β-carotene production profile followed a trend consistent with previous studies by Sharma and Ghoshal (2020) and Ochoa-Viñals et al. (2024), where carotenoid production peaked at intermediate fermentation times, followed by a slight decline, possibly due to product degradation or metabolic shifts during extended cultivation.

The overall fermentation process trends for the three bioreactor replicates over the 10-day period were consistent, as illustrated in the SCADA profiles (Supplementary data, Figures S4–S6). As fermentation progressed, β-carotene concentrations steadily increased (Fig. 3). Notably, the dissolved oxygen (DO) levels in all fermenters decreased to below 15% within the first 24 hours, indicating active growth of *R. glutinis* during the early stages of fermentation (Supplementary Figures S4–S6). The pH of the fermentation medium initially measured 5.5 across all bioreactors and decreased to approximately 4.0 within 24 hours, suggesting rapid utilization of carbohydrates present in the medium. This initial pH drop corresponded with the observed decline in DO levels, further supporting the indication of vigorous microbial activity. The pH remained stable in the range of 4.5 to 5.0 for the remainder of the 10-day fermentation period. Batch-mode fermentation of *R. glutinis*, conducted in triplicate bioreactors, resulted in average β-carotene concentrations of 1 008 µg/g, 1 227 µg/g, and 1 753 µg/g of dry cell weight on days 5, 7, and 10, respectively (Fig. 3). β-carotene production overall is significantly higher in fermenter than bottle (P = 0.0051). Considering the cost of fermentation, it is important to reduce the duration and based on the benchtop bioreactor as well as media bottle validation statistically ideal to stop the fermentation on day 7.

## Nutritional Characterization of Freeze-Dried *R. Glutinis* Fermented Biomass

In addition to β-carotene enrichment, *R. glutinis* fermented sorghum syrup was evaluated for proximate composition and mineral content. The fermented product contained β-carotene as a source of provitamin A, along with crude proteins, crude fats, amino acids, fatty acids, and essential minerals (Supplementary data, Table S4), adding to the overall value proposition of utilizing *R. glutinis* fermentation in a sorghum syrup-based medium.

The protein content of the freeze-dried yeast biomass was approximately 17%, indicating its potential as a protein source for food and feed applications. Interestingly, lipid levels were relatively low, measured at 0.53%, despite *R. glutinis* being widely reported as a high lipid-accumulating yeast (Maza et al., 2020). Additionally, no significant levels of fatty acids or free amino acids were detected in the fermented samples, which may be attributed to the specific fermentation conditions optimized for carotenoid production rather than lipid or protein accumulation.

## Glucosamine

The fermentation process also resulted in the production of glucosamine, measured at 3 073 mg/kg (0.3%) in the *R. glutinis* fermented biomass (Supplementary Table S4). Glucosamine is a critical structural component of fungal cell walls and is widely recognized as a beneficial nutrient for supporting joint health and bone integrity in both humans and animals (Neil et al., 2005). Comparatively, previous studies reported glucosamine levels ranging from 1.91% to 2.4% during *Monascus purpureus* fermentation of corn ethanol coproduct wetcake (Nanjundaswamy and Okeke 2014). Although the glucosamine levels observed here are lower, they demonstrate the potential of *R. glutinis* fermentation to co-produce this value-added compound alongside β-carotene.

## Mineral Composition

Sorghum syrup is inherently rich in minerals such as magnesium, potassium, calcium, phosphorus, zinc, and other trace elements (Eggleston et al., 2022). Minerals play critical roles in both human and animal health, contributing to skeletal structure, enzymatic functions, neuromuscular regulation, oxygen transport, and metabolic processes (Health, 1989; Sampath et al., 2023).

In animal nutrition, mineral supplementation is often necessary due to variations in mineral bioavailability from natural feed sources. Microbial fermentation has been shown to improve mineral bioavailability by converting inorganic minerals into organically chelated forms, which are more stable and better absorbed in the gastrointestinal tract (Chen et al., 2022).

Mineral analysis of *R. glutinis* fermented sorghum syrup confirmed the presence of both macro- and trace minerals (Supplementary Table S4). Notably, potassium (1.39 ± 0.13%), magnesium (1.02 ± 0.07%), and sodium (0.28 ± 0.02%) were present at appreciable levels. The biomass also contained essential trace minerals including iron (54.00 ± 18.24 mg/kg), copper (12.20 ± 2.30 mg/kg), and zinc (22.83 ± 2.89 mg/kg). These findings suggest that *R. glutinis* fermentation not only enhances β-carotene production but may also serve as a platform for generating nutrient-dense, mineral-enriched biomass with potential applications in human and animal nutrition.

## Conclusions

This study demonstrated that sorghum syrup, an agricultural coproduct derived from sweet sorghum processing, is a viable and cost-effective substrate for *R. glutinis* fermentation to produce β-carotene. Process optimization using response surface methodology (RSM) identified the optimal medium composition (9.18% sorghum syrup, 0.13% (NH_4_)₂SO_4_, 0.07% KH₂PO_4_, 0.42% MgSO_4_, and 0.96% yeast extract). Scale-up studies in bioreactors achieved β-carotene yields as high as 1 753 µg/g. In addition to β-carotene, the *R. glutinis* biomass was enriched with protein, glucosamine, and organically chelated minerals, further enhancing its nutritional value. These results indicate that optimized *R. glutinis* fermentation of sorghum syrup can serve as a sustainable approach for producing multiple high-value bioproducts, including www.academic.oup.com/jimbcarotenoids, glucosamine, and mineral-enriched biomass for potential use in food, feed, and nutraceutical industries.

## Supplementary Material

kuaf032_Supplemental_File
