# Supplementary material for: Sustainable β-carotene production from sorghum syrup using Rhodotorula glutinis: bioprocess optimization and scale-up
Source: J Ind Microbiol Biotechnol. 2025 Oct 28;52:kuaf032. doi: 10.1093/jimb/kuaf032 (PMC12631552; doi:10.1093/jimb/kuaf032)
Supplement: kuaf032_Supplemental_File [file kuaf032_supplemental_file.docx]

Supplementary data

Figures


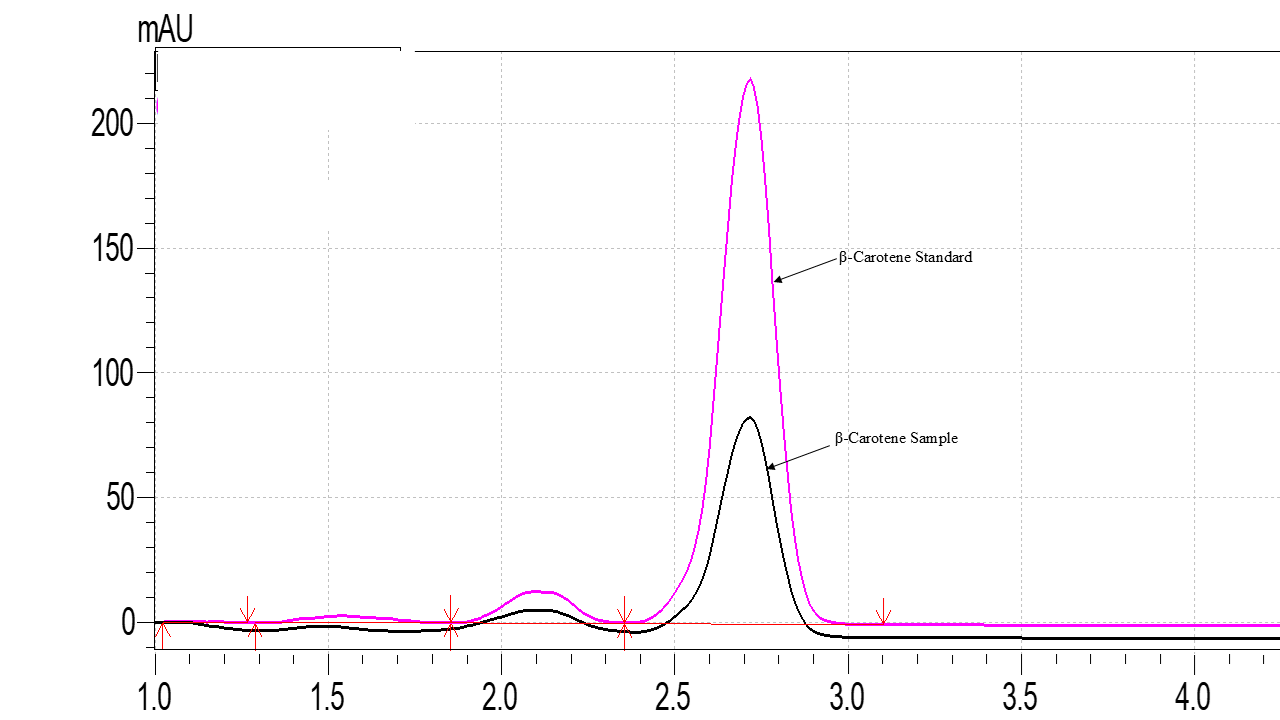


Figure S1. Beta-carotene chromatogram extracted at 455mm.

**
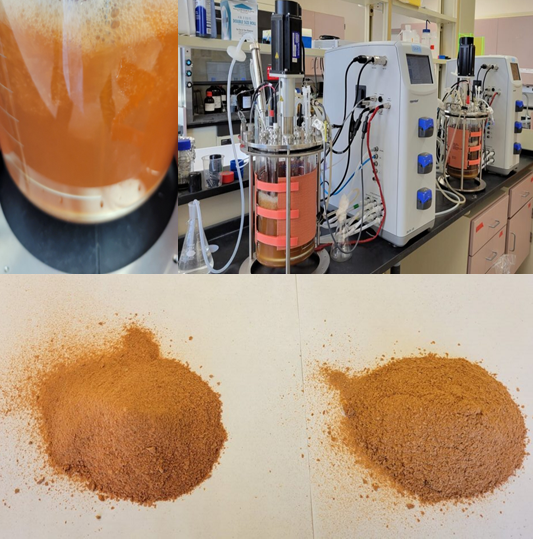
**

Figure S2. Benchtop bioreactor production of beta-carotene from R. glutinis 32766 (top left to right and bottom left). Final freeze-dried yeast cells from bioreactors (bottom right).

**
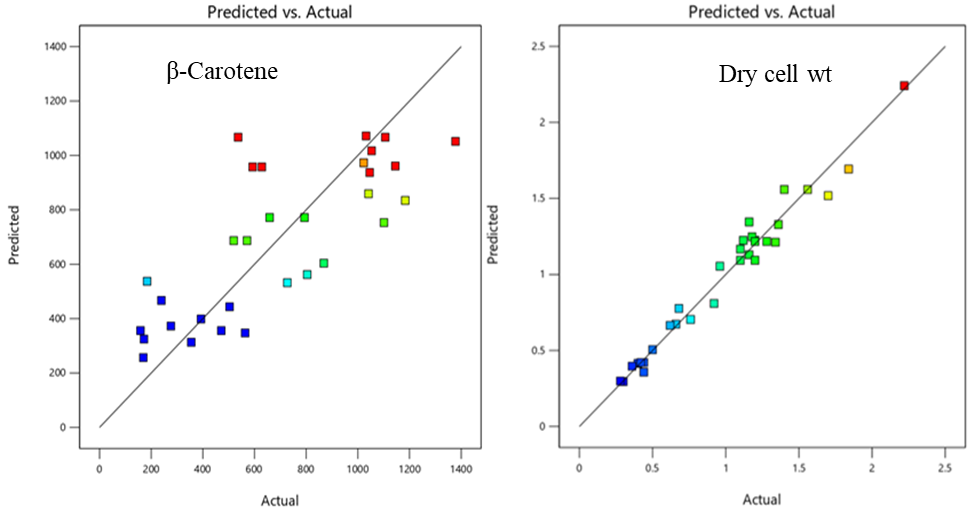
**

*Figure S3. Predicted and actual values of the optimization beta carotene and dry cell wt*


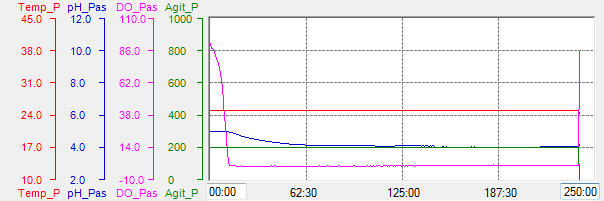


Figure S4.Fermentation profile of three benchtop bioreactors in batch mode for variables like pH, DO, stirring and temperature for fermenter 1


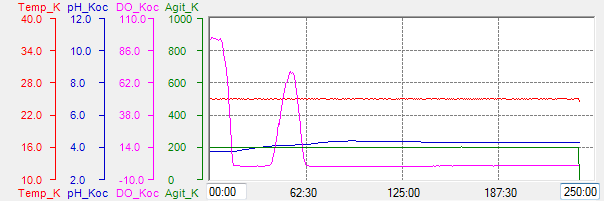


Figure S5.Fermentation profile of three benchtop bioreactors in batch mode for variables like pH, DO, stirring and temperature for fermenter 2


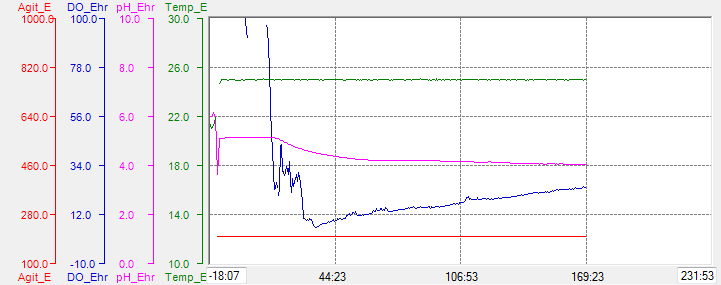


*Figure S6.Fermentation profile of three benchtop bioreactors in batch mode for variables like pH, DO, stirring and temperature for fermenter 3*

*Table S1. Design Matrix from the central composite design (CCD) used during process optimization of the media components for the production of β-carotene from sorghum-fermented R. glutinis.*

| **Run #** | **Sorghum Syrup (g)** | **(NH_4_)_2_SO_4_ (mL)** | **KH_2_PO_4_ (mL)** | **MgSO_4_ (mL)** | **Yeast Extract (g)** |
| --- | --- | --- | --- | --- | --- |
| **1** | 4.83 | 0.15 | 0.01 | 0.26 | 1.00 |
| **2** | 6.72 | 0.05 | 0.10 | 0.43 | 0.28 |
| **3** | 10.00 | 0.50 | 0.01 | 0.10 | 1.00 |
| **4** | 1.00 | 0.30 | 0.10 | 0.38 | 0.56 |
| **5** | 2.58 | 0.50 | 0.06 | 0.41 | 0.10 |
| **6** | 10.00 | 0.27 | 0.06 | 0.26 | 0.10 |
| **7** | 1.00 | 0.05 | 0.04 | 0.27 | 0.46 |
| **8** | 3.39 | 0.22 | 0.04 | 0.50 | 0.73 |
| **9** | 5.19 | 0.23 | 0.05 | 0.10 | 0.62 |
| **10** | 1.00 | 0.50 | 0.01 | 0.50 | 0.87 |
| **11** | 1.00 | 0.30 | 0.10 | 0.38 | 0.56 |
| **12** | 1.00 | 0.50 | 0.09 | 0.10 | 1.00 |
| **13** | 2.08 | 0.50 | 0.04 | 0.24 | 0.63 |
| **14** | 7.93 | 0.50 | 0.01 | 0.36 | 0.37 |
| **15** | 10.00 | 0.13 | 0.03 | 0.50 | 0.74 |
| **16** | 1.00 | 0.05 | 0.10 | 0.10 | 0.10 |
| **17** | 1.00 | 0.05 | 0.09 | 0.50 | 1.00 |
| **18** | 7.17 | 0.43 | 0.08 | 0.42 | 1.00 |
| **19** | 1.00 | 0.42 | 0.01 | 0.10 | 0.10 |
| **20** | 7.17 | 0.43 | 0.08 | 0.42 | 1.00 |
| **21** | 5.19 | 0.23 | 0.05 | 0.10 | 0.62 |
| **22** | 10.00 | 0.05 | 0.01 | 0.10 | 0.10 |
| **23** | 10.00 | 0.05 | 0.10 | 0.15 | 1.00 |
| **24** | 10.00 | 0.50 | 0.03 | 0.10 | 0.10 |
| **25** | 1.65 | 0.40 | 0.08 | 0.12 | 0.10 |
| **26** | 7.93 | 0.50 | 0.10 | 0.10 | 0.33 |
| **27** | 10.00 | 0.27 | 0.06 | 0.26 | 0.10 |
| **28** | 4.83 | 0.15 | 0.01 | 0.26 | 1.00 |
| **29** | 10.00 | 0.50 | 0.10 | 0.50 | 0.10 |
| **30** | 8.49 | 0.05 | 0.10 | 0.48 | 1.00 |
| **31** | 3.25 | 0.21 | 0.01 | 0.50 | 0.10 |

*Table S2. β-carotene concentrations obtained from sorghum syrup fermented R. glutinis 32766 via RSM.*

| **Run #** | **β-carotene conc (µg/g)** | **Cell dry wt (%)** |
| --- | --- | --- |
| **1** | 628.43 | 1.2 |
| **2** | 184.09 | 1.84 |
| **3** | 1045.77 | 1.18 |
| **4** | 658.48 | 0.42 |
| **5** | 503.51 | 0.68 |
| **6** | 471.26 | 1.56 |
| **7** | 868.34 | 0.4 |
| **8** | 1041.49 | 0.92 |
| **9** | 570.95 | 1.12 |
| **10** | 1022.71 | 0.36 |
| **11** | 793.47 | 0.44 |
| **12** | 1053.39 | 0.5 |
| **13** | 1101.09 | 0.76 |
| **14** | 803.96 | 1.1 |
| **15** | 1183.43 | 1.16 |
| **16** | 563.64 | 0.28 |
| **17** | 1031.84 | 0.62 |
| **18** | 536.58 | 1.2 |
| **19** | 172.04 | 0.3 |
| **20** | 1106.12 | 1.1 |
| **21** | 519.50 | 1.2 |
| **22** | 169.43 | 2.22 |
| **23** | 1145.05 | 1.34 |
| **24** | 355.17 | 1.7 |
| **25** | 276.14 | 0.44 |
| **26** | 726.96 | 0.96 |
| **27** | 158.40 | 1.4 |
| **28** | 592.75 | 1.28 |
| **29** | 239.55 | 1.36 |
| **30** | 1378.12 | 1.16 |
| **31** | 392.74 | 0.66 |

### Solutions

Table S3. RSM optimal point prediction for beta-caroene production

| Number | Sorghum Syrup | (NH4)2SO4 | KH2PO4 | MgSO4 | Yeast Extract | Carotenoid | Desirability |  |
| --- | --- | --- | --- | --- | --- | --- | --- | --- |
| 1 | ***9.188*** | ***0.140*** | ***0.071*** | ***0.429*** | ***0.968*** | **1003.379** | **1.000** | **Selected** |
| 2 | *3.385* | *0.216* | *0.037* | *0.500* | *0.730* | 858.989 | 1.000 |  |
| 3 | *7.930* | *0.500* | *0.100* | *0.102* | *0.330* | 532.062 | 1.000 |  |
| 4 | *10.000* | *0.050* | *0.010* | *0.100* | *0.100* | 256.794 | 1.000 |  |
| 5 | *1.000* | *0.303* | *0.100* | *0.384* | *0.564* | 771.643 | 1.000 |  |
| 6 | *1.000* | *0.421* | *0.010* | *0.100* | *0.100* | 324.850 | 1.000 |  |
| 7 | *7.930* | *0.500* | *0.010* | *0.356* | *0.365* | 562.074 | 1.000 |  |
| 8 | *1.000* | *0.500* | *0.089* | *0.100* | *1.000* | 1017.111 | 1.000 |  |
| 9 | *2.575* | *0.500* | *0.063* | *0.410* | *0.100* | 443.484 | 1.000 |  |
| 10 | *2.080* | *0.500* | *0.039* | *0.236* | *0.626* | 753.057 | 1.000 |  |
| 11 | *10.000* | *0.271* | *0.064* | *0.256* | *0.100* | 355.812 | 1.000 |  |
| 12 | *1.000* | *0.050* | *0.036* | *0.270* | *0.465* | 603.738 | 1.000 |  |
| 13 | *4.825* | *0.151* | *0.010* | *0.264* | *1.000* | 957.723 | 1.000 |  |
| 14 | *1.647* | *0.397* | *0.082* | *0.116* | *0.100* | 372.695 | 1.000 |  |
| 15 | *1.000* | *0.500* | *0.010* | *0.500* | *0.865* | 972.776 | 1.000 |  |
| 16 | *1.000* | *0.050* | *0.100* | *0.100* | *0.100* | 347.451 | 1.000 |  |
| 17 | *3.250* | *0.207* | *0.010* | *0.500* | *0.100* | 398.572 | 1.000 |  |
| 18 | *5.185* | *0.230* | *0.054* | *0.100* | *0.622* | 686.745 | 1.000 |  |
| 19 | *1.000* | *0.050* | *0.085* | *0.500* | *1.000* | 1071.618 | 1.000 |  |
| 20 | *10.000* | *0.500* | *0.015* | *0.100* | *1.000* | 937.082 | 1.000 |  |
| 21 | *7.165* | *0.432* | *0.080* | *0.422* | *1.000* | 1066.981 | 1.000 |  |
| 22 | *10.000* | *0.500* | *0.100* | *0.500* | *0.100* | 466.911 | 1.000 |  |
| 23 | *10.000* | *0.050* | *0.100* | *0.148* | *1.000* | 960.937 | 1.000 |  |
| 24 | *10.000* | *0.498* | *0.025* | *0.100* | *0.100* | 313.110 | 1.000 |  |
| 25 | *10.000* | *0.127* | *0.033* | *0.500* | *0.744* | 834.484 | 1.000 |  |
| 26 | *6.715* | *0.050* | *0.096* | *0.432* | *0.280* | 537.242 | 1.000 |  |
| 27 | *4.925* | *0.271* | *0.093* | *0.134* | *0.777* | 835.807 | 1.000 |  |
| 28 | *2.445* | *0.443* | *0.069* | *0.304* | *0.778* | 890.071 | 1.000 |  |
| 29 | *3.220* | *0.223* | *0.044* | *0.248* | *0.731* | 800.524 | 1.000 |  |
| 30 | *2.217* | *0.149* | *0.045* | *0.267* | *0.169* | 407.729 | 1.000 |  |
| 31 | *1.103* | *0.056* | *0.062* | *0.394* | *0.627* | 767.563 | 1.000 |  |
| 32 | *4.110* | *0.271* | *0.098* | *0.310* | *0.300* | 553.118 | 1.000 |  |
| 33 | *9.784* | *0.055* | *0.098* | *0.473* | *0.774* | 886.094 | 1.000 |  |
| 34 | *9.551* | *0.436* | *0.079* | *0.445* | *0.373* | 625.330 | 1.000 |  |
| 35 | *3.472* | *0.469* | *0.095* | *0.369* | *0.183* | 506.379 | 1.000 |  |
| 36 | *1.132* | *0.159* | *0.056* | *0.140* | *0.288* | 470.998 | 1.000 |  |
| 37 | *5.518* | *0.248* | *0.041* | *0.456* | *0.683* | 813.042 | 1.000 |  |
| 38 | *8.504* | *0.190* | *0.026* | *0.349* | *0.601* | 702.381 | 1.000 |  |
| 39 | *6.842* | *0.082* | *0.016* | *0.435* | *0.572* | 692.029 | 1.000 |  |
| 40 | *6.485* | *0.104* | *0.010* | *0.160* | *0.772* | 761.185 | 1.000 |  |
| 41 | *7.304* | *0.113* | *0.041* | *0.474* | *0.545* | 701.256 | 1.000 |  |
| 42 | *5.908* | *0.315* | *0.029* | *0.459* | *0.147* | 436.090 | 1.000 |  |
| 43 | *8.478* | *0.100* | *0.024* | *0.126* | *0.660* | 676.380 | 1.000 |  |
| 44 | *5.805* | *0.200* | *0.095* | *0.481* | *0.521* | 736.639 | 1.000 |  |
| 45 | *7.123* | *0.133* | *0.039* | *0.452* | *0.447* | 628.563 | 1.000 |  |
| 46 | *4.761* | *0.150* | *0.041* | *0.268* | *0.600* | 699.963 | 1.000 |  |
| 47 | *7.811* | *0.139* | *0.057* | *0.130* | *0.479* | 578.596 | 1.000 |  |
| 48 | *8.551* | *0.071* | *0.033* | *0.445* | *0.797* | 856.816 | 1.000 |  |
| 49 | *2.872* | *0.180* | *0.027* | *0.145* | *0.199* | 386.712 | 1.000 |  |
| 50 | *1.274* | *0.133* | *0.078* | *0.120* | *0.601* | 696.707 | 1.000 |  |
| 51 | *2.623* | *0.177* | *0.069* | *0.284* | *0.981* | 999.690 | 1.000 |  |
| 52 | *5.516* | *0.310* | *0.086* | *0.249* | *0.870* | 928.667 | 1.000 |  |
| 53 | *9.622* | *0.079* | *0.019* | *0.446* | *0.213* | 435.766 | 1.000 |  |
| 54 | *2.464* | *0.262* | *0.081* | *0.213* | *0.475* | 644.097 | 1.000 |  |
| 55 | *9.404* | *0.354* | *0.060* | *0.310* | *0.156* | 416.746 | 1.000 |  |
| 56 | *6.016* | *0.389* | *0.029* | *0.258* | *0.595* | 705.406 | 1.000 |  |
| 57 | *1.826* | *0.490* | *0.071* | *0.453* | *0.703* | 884.412 | 1.000 |  |
| 58 | *6.768* | *0.417* | *0.028* | *0.428* | *0.665* | 798.102 | 1.000 |  |
| 59 | *2.347* | *0.052* | *0.077* | *0.195* | *0.558* | 672.923 | 1.000 |  |
| 60 | *2.411* | *0.460* | *0.032* | *0.447* | *0.378* | 623.451 | 1.000 |  |
| 61 | *7.317* | *0.470* | *0.080* | *0.108* | *0.227* | 446.764 | 1.000 |  |
| 62 | *7.260* | *0.426* | *0.051* | *0.241* | *0.182* | 425.965 | 1.000 |  |
| 63 | *2.761* | *0.250* | *0.040* | *0.471* | *0.329* | 577.526 | 1.000 |  |
| 64 | *2.696* | *0.058* | *0.032* | *0.204* | *0.235* | 418.032 | 1.000 |  |
| 65 | *3.795* | *0.249* | *0.086* | *0.130* | *0.373* | 548.409 | 1.000 |  |
| 66 | *7.624* | *0.226* | *0.021* | *0.468* | *0.674* | 787.347 | 1.000 |  |
| 67 | *2.613* | *0.306* | *0.081* | *0.325* | *0.838* | 931.519 | 1.000 |  |
| 68 | *7.272* | *0.096* | *0.063* | *0.480* | *0.565* | 730.016 | 1.000 |  |
| 69 | *7.646* | *0.461* | *0.054* | *0.174* | *0.499* | 635.019 | 1.000 |  |
| 70 | *9.259* | *0.381* | *0.083* | *0.200* | *0.187* | 429.139 | 1.000 |  |
| 71 | *8.907* | *0.492* | *0.010* | *0.243* | *0.471* | 603.288 | 1.000 |  |
| 72 | *3.453* | *0.155* | *0.029* | *0.211* | *0.530* | 632.224 | 1.000 |  |
| 73 | *3.726* | *0.120* | *0.095* | *0.110* | *0.513* | 633.901 | 1.000 |  |
| 74 | *1.677* | *0.257* | *0.052* | *0.473* | *0.949* | 1025.466 | 1.000 |  |
| 75 | *2.923* | *0.392* | *0.044* | *0.372* | *0.899* | 968.653 | 1.000 |  |
| 76 | *9.064* | *0.352* | *0.021* | *0.295* | *0.701* | 769.996 | 1.000 |  |
| 77 | *3.545* | *0.459* | *0.091* | *0.185* | *0.580* | 733.101 | 1.000 |  |
| 78 | *6.105* | *0.290* | *0.073* | *0.484* | *0.390* | 638.932 | 1.000 |  |
| 79 | *7.434* | *0.283* | *0.079* | *0.334* | *0.510* | 683.375 | 1.000 |  |
| 80 | *9.915* | *0.274* | *0.058* | *0.282* | *0.455* | 608.065 | 1.000 |  |
| 81 | *2.264* | *0.223* | *0.027* | *0.104* | *0.558* | 633.984 | 1.000 |  |
| 82 | *4.502* | *0.200* | *0.045* | *0.201* | *0.954* | 938.807 | 1.000 |  |
| 83 | *3.953* | *0.133* | *0.084* | *0.154* | *0.423* | 576.239 | 1.000 |  |
| 84 | *6.532* | *0.469* | *0.049* | *0.312* | *0.650* | 778.300 | 1.000 |  |
| 85 | *4.196* | *0.428* | *0.062* | *0.287* | *0.767* | 865.646 | 1.000 |  |
| 86 | *4.462* | *0.391* | *0.014* | *0.375* | *0.914* | 954.503 | 1.000 |  |
| 87 | *8.724* | *0.255* | *0.043* | *0.328* | *0.262* | 476.977 | 1.000 |  |
| 88 | *2.824* | *0.499* | *0.019* | *0.293* | *0.464* | 637.548 | 1.000 |  |
| 89 | *9.116* | *0.259* | *0.068* | *0.184* | *0.319* | 495.559 | 1.000 |  |
| 90 | *3.741* | *0.347* | *0.090* | *0.340* | *0.875* | 967.228 | 1.000 |  |
| 91 | *8.133* | *0.216* | *0.037* | *0.239* | *0.681* | 741.058 | 1.000 |  |
| 92 | *3.721* | *0.425* | *0.026* | *0.315* | *0.906* | 947.392 | 1.000 |  |
| 93 | *9.408* | *0.427* | *0.085* | *0.342* | *0.415* | 631.391 | 1.000 |  |
| 94 | *1.361* | *0.486* | *0.017* | *0.459* | *0.681* | 834.859 | 1.000 |  |
| 95 | *3.900* | *0.278* | *0.059* | *0.107* | *0.242* | 434.826 | 1.000 |  |
| 96 | *2.712* | *0.148* | *0.026* | *0.307* | *0.196* | 422.285 | 1.000 |  |
| 97 | *5.348* | *0.253* | *0.085* | *0.245* | *0.160* | 423.374 | 1.000 |  |
| 98 | *8.051* | *0.243* | *0.093* | *0.406* | *0.173* | 469.731 | 1.000 |  |
| 99 | *7.305* | *0.426* | *0.073* | *0.369* | *0.211* | 494.574 | 1.000 |  |
| 100 | *9.003* | *0.430* | *0.056* | *0.170* | *0.446* | 591.079 | 1.000 |  |

Table S4. Nutritional analyses profile of R. glutinis (ATCC 32766) fermented in a sorghum syrup-based media.

| **Nutritional Profile ( Units are next to the nutrients)** | **Range of nutrient (with Std Err)** |
| --- | --- |
| ***Fatty acids*** |  |
| Palmitic (C16:0) g/100g | 0.07±0.03 |
| Stearic (C18:0) g/100g | 0.02±0.01 |
| Oleic (C18:1, Cis) g/100g | 0.34±0.14 |
| Linoleic (C18:2, Trans) g/100g | 0.05±0.03 |
| alpha-Linoleic (C18:3, alpha) g/100g | 0.01±0.1 |
| Lignoceric (C24:0) g/100g | 0.01±0.01 |
| Saturated fat total g/100g | 0.11±0.05 |
| Polyunsaturated fat (total) g/100g | 0.06±0.04 |
| Monounsaturated fats (total) g/100g | 0.35±0.14 |
| Omega 3 fatty acids (total) g/100g | 0.01±0.01 |
| Omega 6 fatty acids (total) g/100g | 0.05±0.03 |
| Omega 9 fatty acids (total) g/100g | 0.34±0.14 |
| Glucosamine (HCL total) mg/kg | 3073±900 |
| ***Other biochemicals*** |  |
| Crude protein % | 17.2±1.5 |
| Crude fats % | 0.53±0.23 |
| Cysteine % | 0.13±0.02 |
| Methionine % | 0.2±0.02 |
| Tryptophan % | 0.15±0.02 |
| ***Minerals*** |  |
| Sulphur (total) % | 1.67±0.14 |
| Phosphorus (total) % | 0.55±0.04 |
| Potassium (total) % | 1.39±0.13 |
| Magnesium (total) % | 1.02±0.07 |
| Calcium (total) % | 0.16±0.01 |
| Sodium (total) % | 0.28±0.02 |
| Iron (total) ppm | 54±18.24 |
| Manganese (total) ppm | 8.83±0.85 |
| Copper (total) ppm | 12.2±2.3 |
| Zinc (total) ppm | 22.83±2.89 |
| Sulphur (total) % | 1.67±014 |
| Phosphorus (total) % | 0.55±0.04 |
| Potassium (total) % | 1.39±0.13 |
